# Fecal microbiota transplantation improves chicken growth performance by balancing jejunal Th17/Treg cells

**DOI:** 10.1186/s40168-023-01569-z

**Published:** 2023-06-21

**Authors:** Ziyu Ma, Muhammad Akhtar, Hong Pan, Qiyao Liu, Yan Chen, Xinxin Zhou, Yingting You, Deshi Shi, Huazhen Liu

**Affiliations:** 1 Key Laboratory of Agricultural Animal Genetics, Breeding and Reproduction of Ministry of Education, Huazhong Agricultural University, Wuhan, 430070 People’s Republic of China; 2 Department of Preventive Veterinary Medicine, College of Animal Science and Veterinary Medicine, Huazhong Agricultural University, Wuhan, 430070 People’s Republic of China

**Keywords:** Fecal microbiota transplantation, Th17/Treg cell balance, Chicken growth performance, *Lactobacillus*, Tryptophan

## Abstract

**Background:**

Intestinal inflammation has become a threatening concern in chicken production worldwide and is closely associated with Th17/Treg cell imbalance. Several studies described that gut microbiota is significantly implicated in chicken growth by modulating intestinal immune homeostasis and immune cell differentiation. Whether reshaping gut microbiota by fecal microbiota transplantation (FMT) could improve chicken growth by balancing Th17/Treg cells is an interesting question.

**Results:**

Here, the chickens with significantly different body weight from three different breeds (Turpan cockfighting × White Leghorn chickens, white feather chickens, and yellow feather chickens) were used to compare Th17 and Treg cells. qPCR and IHC staining results indicated that Th17 cell-associated transcriptional factors *Stat3* and *rorγt* and cytokines *IL-6*, *IL-17A*, and *IL-21* were significantly (*P* < 0.05) higher in the jejunum of low body weight chickens, while Treg cell-associated transcriptional factor *foxp3* and cytokines *TGF-β* and *IL-10* were significantly (*P* < 0.05) lower in the jejunum of low body weight chickens, indicating imbalanced Th17/Treg cells were closely related to chicken growth performance. Transferring fecal microbiota from the healthy donor with better growth performance and abundant *Lactobacillus* in feces to 1-day-old chicks markedly increased growth performance (*P* < 0.001), significantly decreased Th17 cell-associated transcriptional factors and cytokines, and increased Treg cell-associated transcriptional factors and cytokines in the jejunum (*P* < 0.05). Furthermore, FMT increased the abundance of *Lactobacillus* (FMT vs Con; 84.98% vs 66.94%). Besides, the metabolites of tryptophan including serotonin, indole, and 5-methoxyindoleacetate were increased as well, which activated their receptor aryl-hydrocarbon-receptor (*AhR*) and expressed more *CYP1A2* and *IL-22* to maintain Th17/Treg cell balance and immune homeostasis.

**Conclusion:**

These findings suggested that imbalanced Th17/Treg cells decreased chicken growth performance, while FMT-reshaped gut microbiota, i.e., higher *Lactobacilli*, increased chicken growth performance by balancing Th17/Treg cells.

Video Abstract

**Supplementary Information:**

The online version contains supplementary material available at 10.1186/s40168-023-01569-z.

## Introduction

Chronic intestinal inflammation decreases feed intake and nutrient digestion and absorption and dysregulates the commensal balance, barrier permeability, mucosal structural physiology, immune response, and homeostasis, resulting in a decline in chicken growth performance [1–3]. How to decrease intestinal inflammation and improve chicken growth performance has become a special concern in chicken production worldwide [4]. There are many immune cells in the intestine such as helper T (Th) cells and regulatory T (Treg) cells, which work together to keep the balance of immune response. Otherwise, breaking this balance will result in intestinal inflammation [5]. Intestinal immune cells produced more pro-inflammatory cytokines, i.e., interleukin *(IL)-6* and *IL-1β*, and less anti-inflammatory cytokines, i.e., *IL-10* and transforming growth factor-β (*TGF-β*) in the chicken intestine during chronic intestinal inflammation [6–8]. Other studies indicated that pro-inflammatory cytokines were mainly produced by Th cells, especially Th17 cells, while anti-inflammatory cytokines were mainly produced by Treg cells [9, 10]. Furthermore, it has been established that chronic intestinal inflammation is closely associated with the imbalanced Th17 and Treg cells through critical transcriptional factors such as retinoid-related orphan receptor gamma t (*rorγt*) and forkhead box P3 (*foxp3*) and key cytokines such as *IL-6*, *IL-1β*, *IL-17A*, *IL-17F*, *IL-21*, *IL-10*, and *TGF-β* [11–13]. For example, *IL-6* causes signal transducer and activator of transcription 3 (*Stat3*) phosphorylation, induces *rorγt*, and promotes Th17 cell differentiation through *IL-23R*-dependent *Stat3* signaling, which exacerbates intestinal inflammation [14, 15]. Chronic intestinal inflammation-mediated *IL-1β* secretion also promotes Th17 cell accumulation, which produces *IL-17A* and *IL-17F* and worsens intestinal inflammation [16]. Likewise, *IL-21* also depends on the *Stat3* signaling for Th17 cell differentiation and also inhibits Treg cells’ functions, thus facilitating Th17 cell differentiation in intestinal inflammation [15, 17]. On the other hand, *TGF-β* stimulates naive CD4^+^ T cells and induces the activation of *foxp3* transcription, which subsequently facilitates Treg cell differentiation that inhibits other T-cell activation and controls inflammation by regulating immune response [14, 18]. In our previous research work, the inflammation levels in the duodenum, jejunum, ileum, and cecum between high body weight and low body weight chickens were compared, and we found that the inflammation level in the jejunum is significantly different with higher expression of *IL-10*, *IL-4*, *TGF-β*, and lower expression of *IL-1β*, *IFN-γ*, and *TNF-α* in high body weight chickens (unpublished data), suggesting that decreased inflammation level in the jejunum by increasing Treg cell-associated cytokines is closely related to chicken growth performance. Besides, jejunum has unique features with the largest surface area for efficient nutrient absorption/intake; if inflammation destroyed its structure, the growth performance would substantially be affected [2]. Therefore, balancing Th17/Treg cells in jejunum is critical for improving chicken growth performance.

Gut microbiota is closely related to intestinal immune homeostasis and significantly contributes to preventing intestinal inflammation [19, 20]. Further studies indicated that gut microbiota could regulate immune homeostasis by balancing Th17/Treg cells [21, 22]. For instance, *Bacteroides fragilis* and *Bifidobacterium infantis* trigger Treg cells by increasing *foxp3* expression [14, 23], and *Lactobacillus plantarum* regulates Th17/Treg cell balance in the immunosuppressed mice [24], maintaining host intestinal homeostasis. It has been reported that fecal microbial transplantation (FMT) is an effective way to reshape gut microbiota [25]. For instance, FMT could enhance microbial diversity and richness [26] and improve chicken growth performance [27]. It has also been reported that established gut microbiota could improve chicken growth performance by mitigating intestinal inflammation [2]. Therefore, whether FMT could improve chicken growth performance by balancing Th17/Treg cells has become an interesting question.

To answer this question, firstly, the chickens with significantly different growth performance from three different chicken breeds (Turpan cockfighting × White Leghorn chickens, white feather chickens, and yellow feather chickens) were used in the current study to elucidate the association of decreased growth performance with imbalanced Th17/Treg cells. Secondly, to verify whether early colonization of gut microbiota could improve growth performance by maintaining Th17/Treg cell balance, transplanting fecal microbiota from healthy donor chickens with better growth performance to 1-day-old chicks was accomplished. In addition, how FMT improved growth performance by balancing Th17/Treg cells was investigated as well.

## Results

### The growth performance of high and low body weight chickens was significantly different

**Fig. 1:**
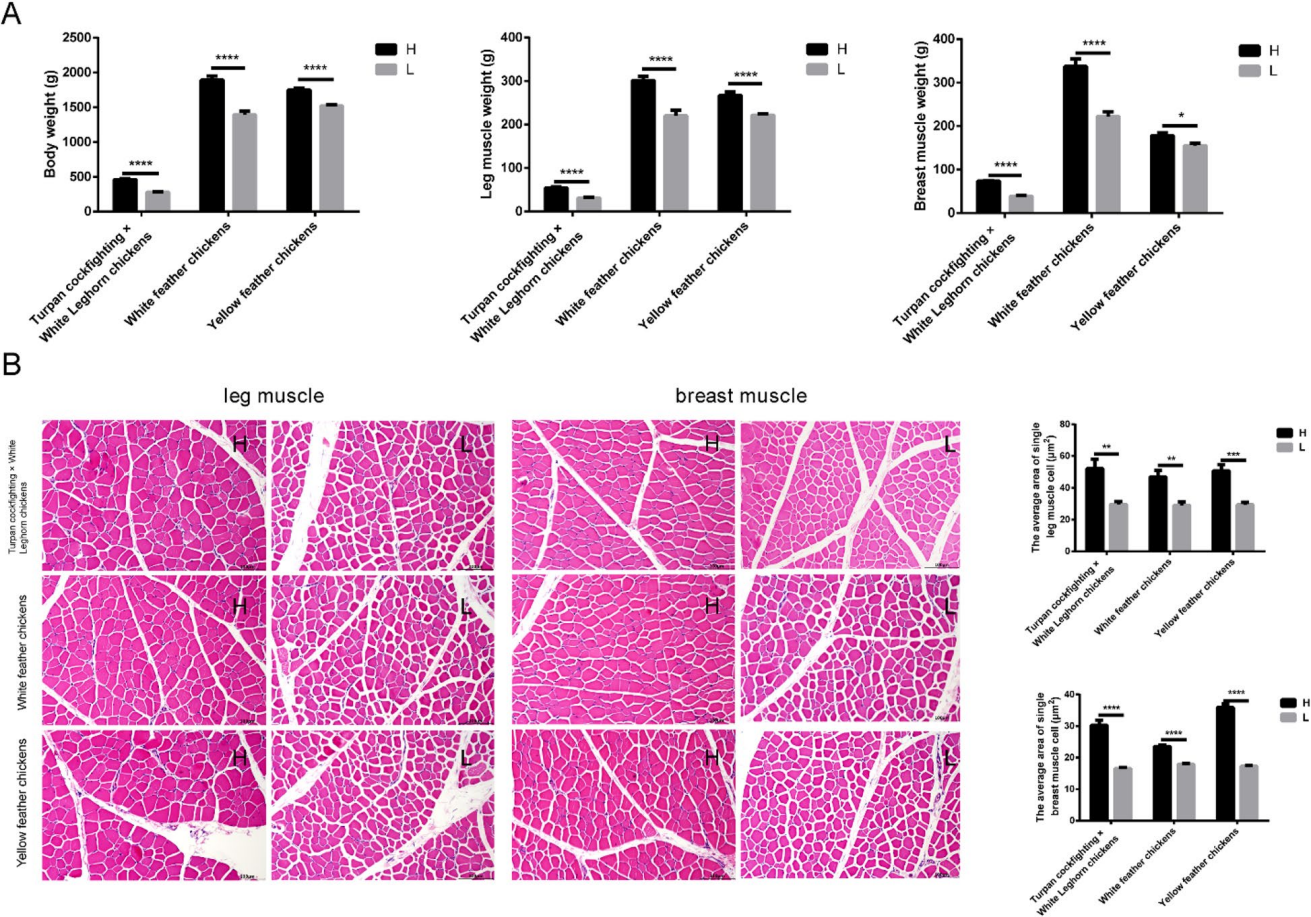
Differences in growth performance between high and low body weight chickens from three different breeds (Turpan cockfighting × White Leghorn chickens, white feather chickens, and yellow feather chickens). All chickens from three different breeds (Turpan cockfighting × White Leghorn chickens, white feather chickens, and yellow feather chickens) were grown for 6 weeks. **A** The comparison of body weight, leg muscle weight, and breast muscle weight between high and low body weight chickens. **B** The comparison of the single leg and breast muscle cell’s cross-sectional area between high and low body weight chickens (H & E staining). Scale bars, 100 μm. Data are shown as mean ± SEM. *P < 0.05, **P < 0.01, ***P < 0.001, ****P < 0.0001. H, high body weight group (*n* = 10); L, low body weight group (*n* = 10)

In the present study, three different chicken breeds (Turpan cockfighting × White Leghorn chickens, white feather chickens, and yellow feather chickens) were used to get the individuals with significantly different growth performances, respectively. The body weight (*P* < 0.0001), leg muscle weight (*P* < 0.01), and breast muscle weight (*P* < 0.01) were significantly higher in high body weight groups than that in low body weight groups in all three breeds (Fig. 1A). Furthermore, hematoxylin and eosin (HE) staining results showed that the single leg (*P* < 0.01) and breast (*P* < 0.0001) muscle cell’s cross-sectional area was significantly larger in high body weight group compared with the low body weight group in all three breeds (Fig. 1B). These results indicated that chickens of three different breeds grown under the similar rearing conditions exhibited significantly differential growth performance.

### Imbalanced Th17/Treg cells significantly decreased chicken growth performance

**Fig. 2:**
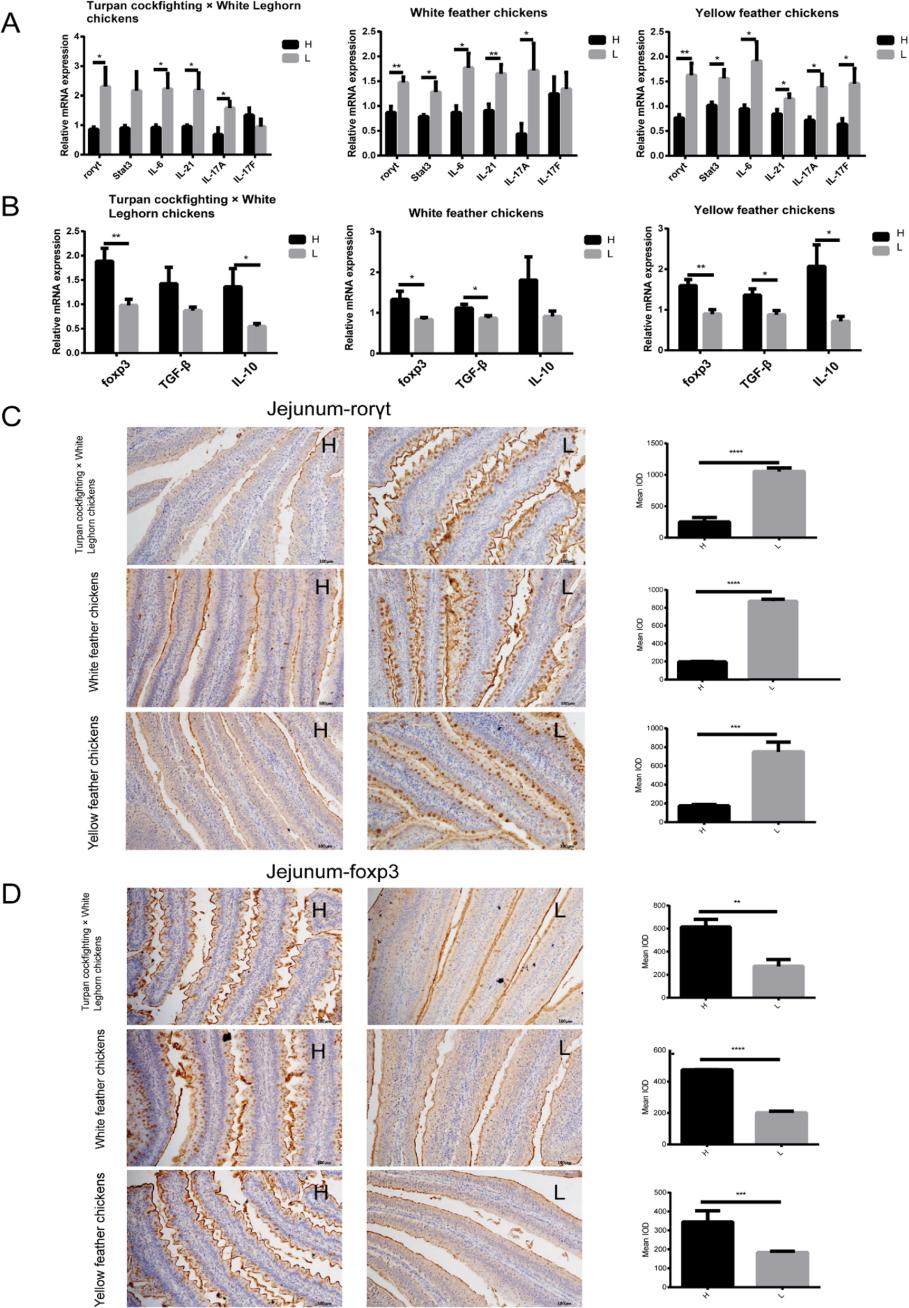
Comparison of Th17/Treg cell-associated transcriptional factors and cytokine's expression in jejunum between high and low body weight chickens from three different breeds (Turpan cockfighting × White Leghorn chickens, white feather chickens, and yellow feather chickens). **A** The comparison of relative mRNA expressions of Th17 cell-related transcriptional factors and cytokines in the jejunum of low and high body weight chickens (qPCR). **B** The comparison of relative mRNA expressions of Treg cell-related transcriptional factors and cytokines in the jejunum of low and high body weight chickens (qPCR). **C** The comparison of protein expression levels of *rorγt* in the jejunum low and high body weight chickens (immunohistochemical staining). **D** The comparison of protein expression levels of *foxp3* in the jejunum of low and high body weight chickens (immunohistochemical staining). Scale bars, 100 μm. Data are shown as mean ± SEM. *P < 0.05, **P < 0.01, ***P < 0.001, ****P < 0.0001. IOD, integrated optical density; H, high body weight group (*n* = 10); L, low body weight group (*n* = 10)

An imbalance of immune cells usually leads to intestinal inflammatory disease, which reduces growth performance [28]. Th17 and Treg cells are very important immune cells in the intestine. Therefore, the transcriptional factors and cytokines associated with Th17 and Treg cells in jejunum were compared between high and low body weight chickens. At the mRNA level, the relative expressions of Th17 cell-associated retinoid-related orphan receptor gamma t (*rorγt*); interleukin (*IL*)-*6*, *IL-17A*, and *IL-21*; and signal transduction and activators of transcription 3 (*Stat3*) were significantly (*P* < 0.05) higher in low body weight group compared with high body weight group (Fig. 2A). However, the relative mRNA expressions of Treg cell-associated forkhead box P3 (*foxp3*), transforming growth factor-β (*TGF-β*) and interleukin-10 (*IL-10*), were significantly (*P* < 0.05) lower in low body weight group than that in high body weight group (Fig. 2B). At the protein level, the expression of *rorγt* was significantly (*P* < 0.001) higher (Fig. 2C), yet the expression of *foxp3* was significantly (*P* < 0.01) lower (Fig. 2D) in low body weight chickens of all three breeds. The above results indicated that the increased Th17 cells and decreased Treg cells reduced chicken growth performance.

### Fecal microbiota transplantation could significantly improve chicken growth performance and balance Th17/Treg cells

**Fig. 3:**
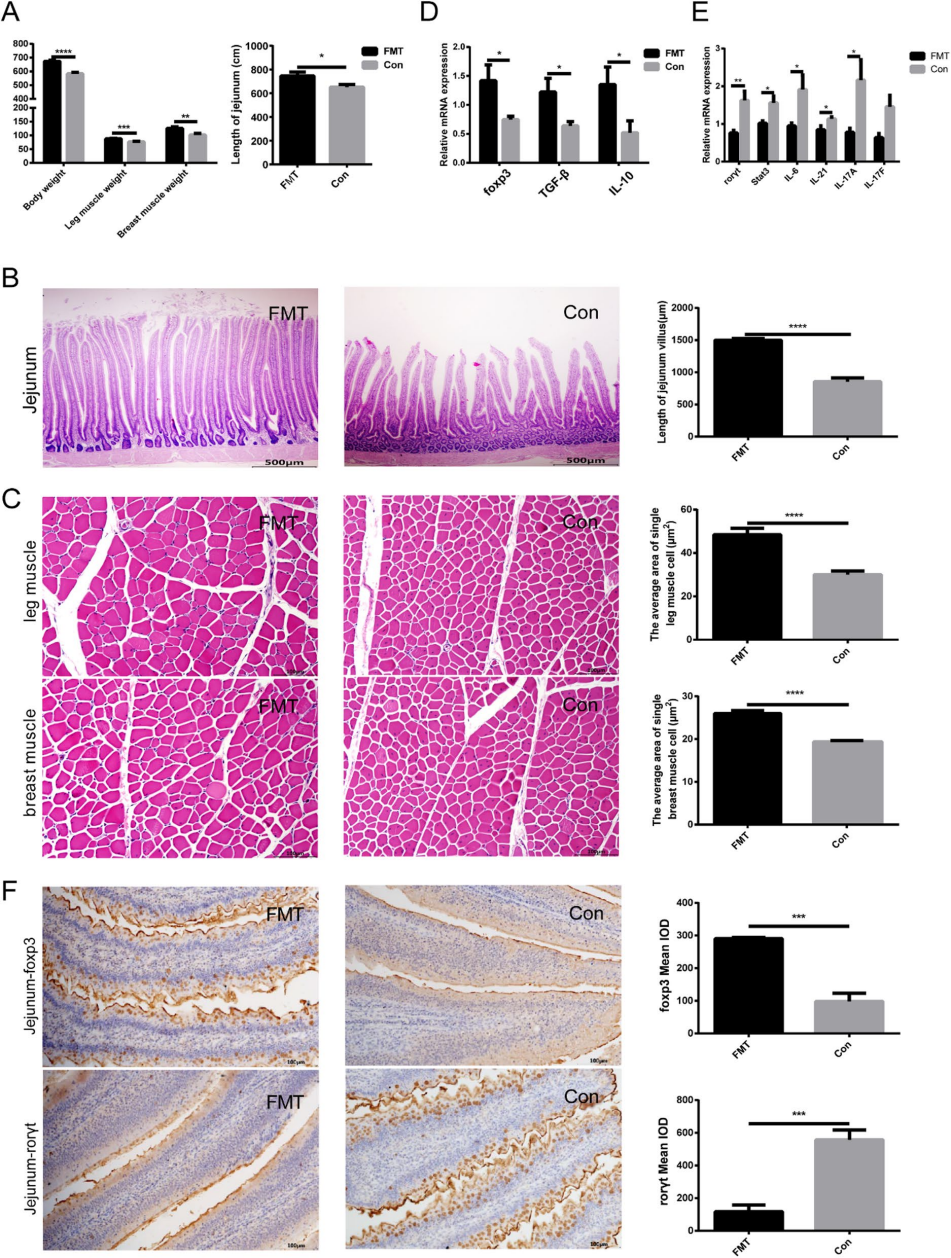
Effects of fecal microbiota transplantation (FMT) on growth performance and Th17/Treg cell-related factors in the jejunum of chickens. **A** The comparison of body weight, leg muscle weight, breast muscle weight, and jejunum length between the FMT and control groups. **B** The comparison of length of jejunal villus between the FMT and control groups (H & E staining). Scale bars, 500 μm. **C** The comparison of single leg and breast muscle cell’s cross-sectional area between the FMT and control groups (H & E staining). Scale bars, 100 μm. **D** The comparison of relative mRNA expression of Treg cell-related factors in the jejunum of FMT and control groups (qPCR). **E** The comparison of relative mRNA expression of Th17 cell-related factors in the jejunum of FMT and control groups (qPCR). **F** The comparison of protein expression level of *foxp3* and *rorγt* in the jejunum of FMT and control groups (immunohistochemical staining). Scale bars, 100 μm. IOD, integrated optical density. Data were shown as mean ± SEM. *P < 0.05, **P < 0.01, ***P < 0.001, ****P < 0.0001. FMT, fecal microbiota transplantation group (*n* = 10); Con, control group (*n* = 10)

In order to investigate whether reshaping gut microbiota could improve chicken growth performance by balancing Th17/Treg cells, fecal microbiota transplantation (FMT) experiments were conducted. The results showed that the body weight (FMT vs Con, 627.40 ± 7.35 g vs 567.30 ± 3.89 g, *P* < 0.0001), leg muscle weight (FMT vs Con, 41.33 ± 0.55 g vs 37.07 ± 0.66 g, *P* < 0.001), and breast muscle weight (FMT vs Con, 61.23 ± 1.68 g vs 51.44 ± 1.50 g, *P* < 0.01) were significantly higher in FMT group compared with the control group (Fig. 3A). The jejunum length (FMT vs Con, 745.3 ± 32.79 cm vs 650.9 ± 22.17 cm) of the FMT group was also observed significantly (*P* < 0.05) longer than that of the control group (Fig. 3A). HE staining results indicated that the length of jejunal villi (FMT vs Con, 1499 ± 26.87 μm vs 853.6 ± 58.82 μm, *P* < 0.0001) was significantly larger in FMT group compared with the control group (Fig. 3B), and the mean cross-sectional area of single leg muscle cell (FMT vs Con, 48.45 ± 2.92 μm^2^ vs 29.99 ± 1.66 μm^2^, *P* < 0.0001) and single breast muscle cell (FMT vs Con, 25.99 ± 0.69 μm^2^ vs 19.37 ± 0.31 μm^2^, *P* < 0.0001) was also significantly larger in FMT group (Fig. 3C). Furthermore, the relative mRNA expression of Treg cell-related transcriptional factors and cytokines such as *foxp3* (FMT vs Con, 1.41 ± 0.27 vs 0.79 ± 0.07, *P* < 0.05), *TGF-β* (FMT vs Con, 1.22 ± 0.24 vs 0.64 ± 0.073, *P* < 0.05), and *IL-10* (FMT vs Con, 1.46 ± 0.27 vs 0.58 ± 0.17, *P* < 0.05) was significantly higher in FMT group (Fig. 3D), while Th17 cell-associated transcriptional factors and cytokines such as *rorγt* (FMT vs Con, 0.82 ± 0.14 vs 1.87 ± 0.20, *P* < 0.01), *Stat3* (FMT vs Con, 0.84 ± 0.12 vs 1.53 ± 0.26, *P* < 0.05), *IL-6* (FMT vs Con, 0.66 ± 0.13 vs 1.62 ± 0.39, *P* < 0.05), *IL-17A* (FMT vs Con, 0.78 ± 0.11 vs 2.16 ± 0.54, *P* < 0.05), and *IL-21* (FMT vs Con, 0.85 ± 0.10 vs 1.136 ± 0.05, *P* < 0.05) were significantly lower in FMT group (Fig. 3E). Moreover, at protein level, IHC results indicated that the expression of *foxp3* (FMT vs Con, 290.60 ± 2.05 vs 98.33 ± 14.30, *P* < 0.001) was significantly higher in FMT group, while the expression of *rorγt* (FMT vs Con, 117.70 ± 23.38 vs 557.00 ± 34.50, *P* < 0.001) was significantly lower in FMT group (Fig. 3F). The above results indicated that FMT could significantly improve chicken growth performance and decrease Th17 cells yet increase Treg cells in the jejunum.

### Fecal microbiota transplantation significantly increased the abundance of *Lactobacillus* in the jejunum

**Fig. 4:**
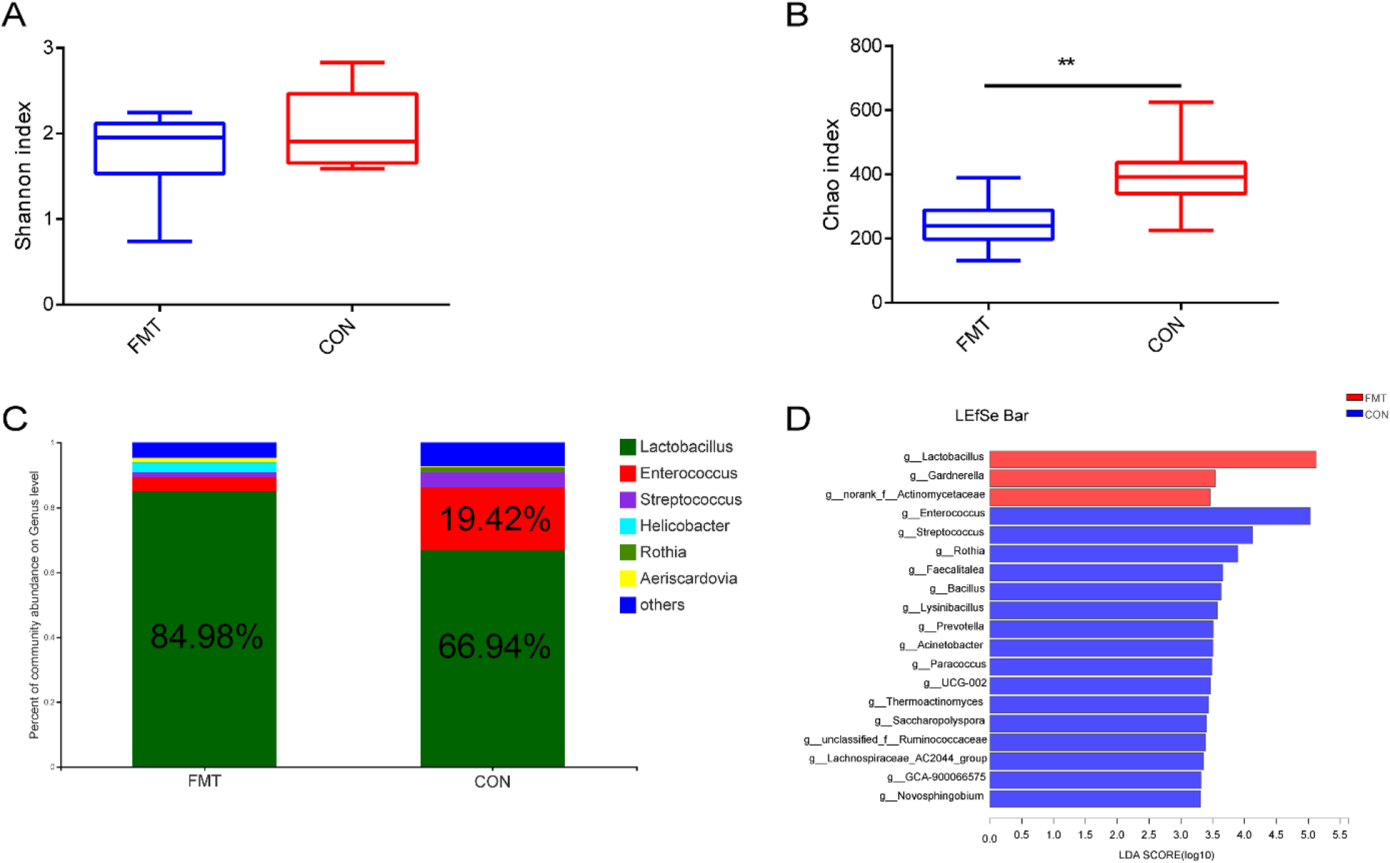
Effects of fecal microbiota transplantation (FMT) on the microbial communities in the jejunum of recipient chickens. **A** The comparison of jejunal microbiota diversity (Shannon index) between the FMT and control groups. **B** The comparison of jejunal microbiota richness (Chao index) between the FMT and control groups. **C** At the genus level, the comparison of relative abundance of *Lactobacillus* between the FMT and control groups. **D** The comparison of relative abundance of *Lactobacillus*, *Gardnerella*, *g_norank_f_*Actinomycetaceae, and other bacteria between the FMT and control groups. LDA score ≥ 2. **P < 0.01. FMT, fecal microbiota transplantation group (*n* = 10); Con, control group (*n* = 10)

To elucidate whether FMT improved chicken growth performance and balanced Th17/Treg cells by reshaping gut microbiota, 16S rRNA gene sequencing was conducted to compare the bacterial community composition in the jejunal contents of the FMT and control groups. The microbial diversity (Shannon index) was prominently lower, although non-significant in FMT group (Fig. 4A). However, the microbial abundance (Chao index) was significantly (*P* < 0.01) lower in the FMT group compared with the control group (Fig. 4B), indicating that microbiota transplantation might have distinctly altered the microbial community diversity. At the genus level, the relative abundance of *Lactobacillus* was higher in the FMT group (FMT vs Con, 84.98% vs 66.94%), while the relative abundance of opportunistic pathogens, i.e., *Enterococcus* (FMT vs Con, 4.42% vs 19.42%) and *Streptococcus* (FMT vs Con, 1.53% vs 4.58%), was lower in the FMT group compared with the control group (Fig. 4C). Furthermore, differential analysis of bacterial communities at the genus level, i.e., the linear discriminant analysis effect size (LEfSe), showed that the relative abundance of some bacteria such as *Lactobacillus*, *Gardnerella*, and g_norank_f_Actinomycetaceae was higher in the FMT group, while the relative abundance of some opportunistic pathogenic bacteria such as *Enterococcus* and *Streptococcus* was higher in the control group compared with the FMT group (Fig. 4D). The sequencing results indicated that FMT increased the abundance of *Lactobacillus* while reduced the abundance of opportunistic pathogenic bacteria.

### Increased *Lactobacillus* produced more metabolites in the tryptophan signal pathway

**Fig. 5:**
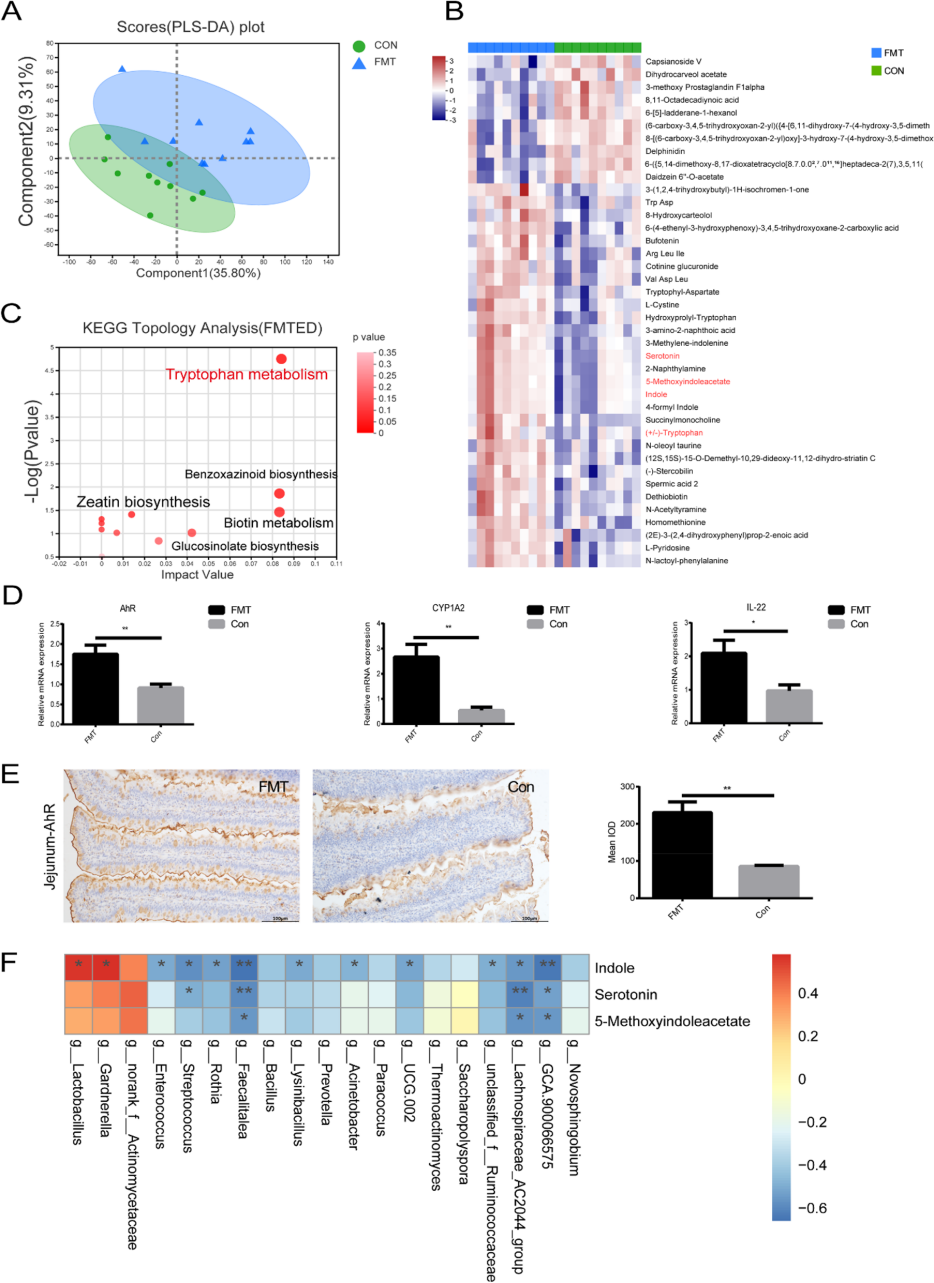
Fecal microbiota transplantation (FMT) markedly alters the metabolite profile in the jejunum of recipient chickens. **A** The comparison of metabolomics profile of jejunal contents between the FMT and control groups (partial least-squares discriminant analysis). **B** The comparison of heat map of 40 different metabolites between the FMT and control groups. **C** The comparison of metabolite pathway enrichment analysis between the FMT and control groups. **D** The comparison of relative mRNA expression of tryptophan metabolite receptor aryl hydrocarbon receptor (*AhR*), *CYP1A2*, and *IL-22* between the FMT and control groups (qPCR). **E** The comparison of protein expression level of aryl hydrocarbon receptor (*AhR*) between the FMT and control groups (immunohistochemical staining). Scale bars, 100 μm. **F** Heat map of Spearman’s correlations between differential jejunal microbiota and tryptophan metabolites. Data were shown as mean ± SEM. *P < 0.05, **P < 0.01. FMT, fecal microbiota transplantation group (*n* = 10); Con, control group (*n* = 10). Metabolites with the variable importance in the projection (VIP) value of the PLS-DA model > 1

It has been established that *Lactobacillus* improves chicken growth performance by producing special metabolites. Liquid chromatography-tandem mass spectrometry (LC–MS) was performed to compare the metabolite enrichments in jejunal content between the FMT and control groups. The partial least-squares discrimination analysis (PLS-DA) model showed a significant separation in the metabolites between the two groups (*Q*^2^ > 0.5) (Fig. 5A). Among the top 40 prominently (*P* < 0.05) different metabolites based on the variable importance in the projection (*VIP* > 1), tryptophan and its three major tryptophan metabolites including serotonin, indole, and 5-methoxyindoleacetate were significantly (*P* < 0.05) enriched in the jejunal contents of FMT group (Fig. 5B). KEGG functional analysis further indicated that tryptophan metabolism pathway has significantly (*P* < 0.0001) higher enrichment and impact compared with the benzoxazinoid biosynthesis, zeatin biosynthesis, biotin metabolism, and glucosinolate biosynthesis pathways (Fig. 5C). As tryptophan metabolite receptor, aryl hydrocarbon receptor (*AhR*) signaling pathway, is implicated in ameliorating intestinal inflammation through balancing Th17/Treg cells, so we examined the expression levels of *AhR* receptor and its response genes cytochrome P450, family 1, subfamily A, polypeptide 2 (*CYP1A2*), and *IL-22* in the jejunum of both groups. We found that the relative mRNA expressions of *AhR* (FMT vs Con, 1.75 ± 0.22 vs 0.91 ± 0.09, *P* < 0.01), *CYP1A2* (FMT vs Con, 2.66 ± 0.50 vs 0.54 ± 0.13, *P* < 0.01), and *IL-22* (FMT vs Con, 2.09 ± 0.39 vs 0.97 ± 0.18, *P* < 0.05) were significantly higher in the FMT group compared with the control group (Fig. 5D). Similarly, IHC results showed that the protein expression of *AhR* (FMT vs Con, 230.30 ± 28.87 vs 85.33 ± 2.91, *P* < 0.01) was also significantly higher in the FMT group (Fig. 5E). Spearman correlation analysis indicated that the relative abundance of *Lactobacillus* was positively correlated with serotonin, indole, and 5-methoxyindoleacetate (*P* < 0.05) (Fig. 5F). These results indicated that a higher *Lactobacillus* abundance promoted tryptophan metabolism and regulated intestinal metabolites by modulating *AhR* metabolic pathway, which in turn could maintain Th17/Treg cell balance in the jejunum of FMT chickens.

### Increased metabolites in the tryptophan signal pathway were closely related to the balance of Th17/Treg cells

**Fig. 6:**
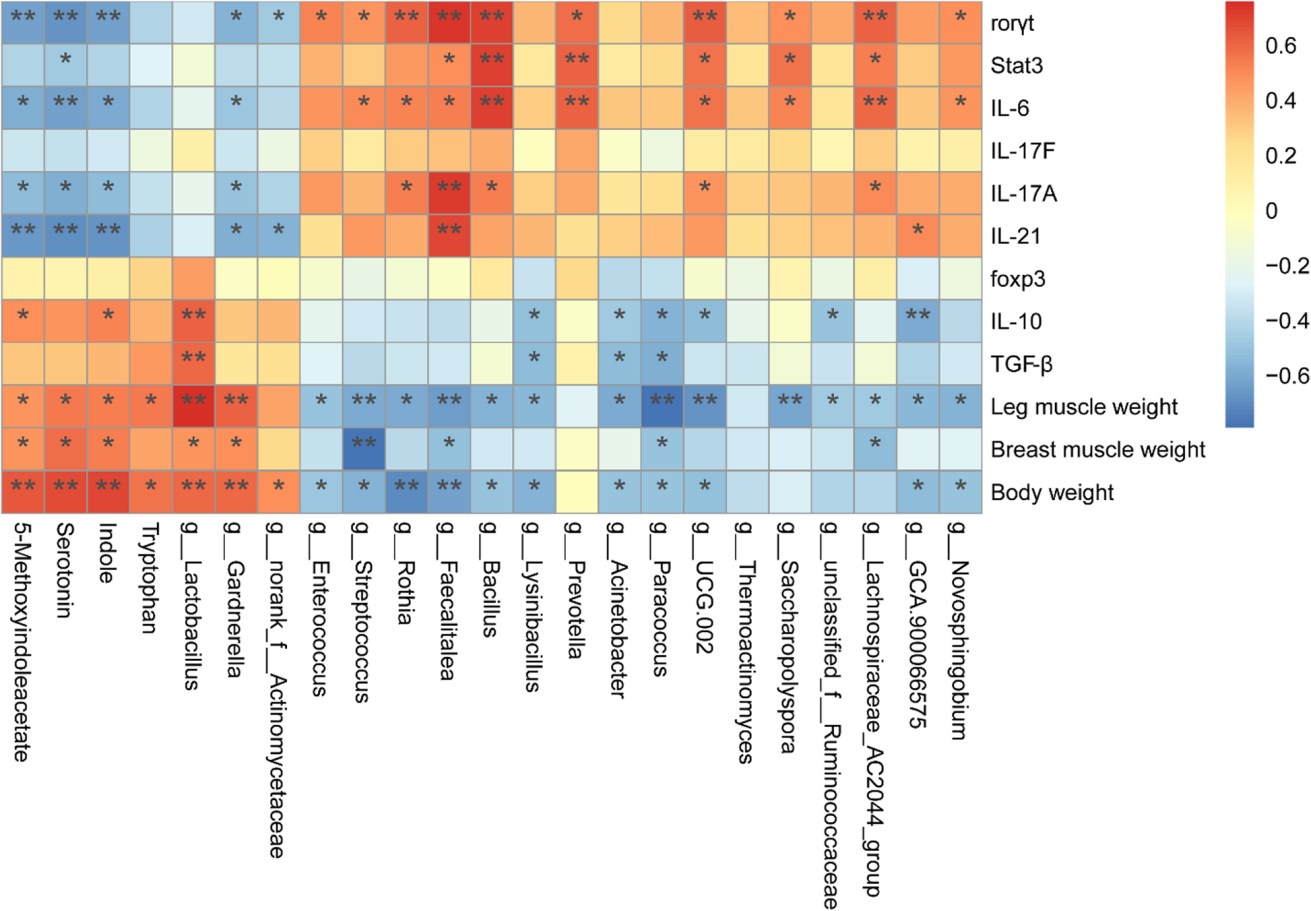
Heatmap of Spearman’s correlations between differential jejunal microbiota, tryptophan metabolites and Th17/Treg cell-related transcriptional factors and cytokines, and growth performance. The colors range from blue (negative correlation) to red (positive correlation). *P < 0.05 and **P < 0.01

Spearman correlation analysis was used to investigate the relationship between tryptophan metabolites, jejunal microbiota, Th17/Treg-related transcriptional factors, and cytokines and growth performance. The analysis showed that the relative abundance of *Lactobacillus*, serotonin, indole, and 5-methoxyindoleacetate tryptophan metabolites was positively correlated with growth performance and Treg cell-related transcriptional factors and cytokines such as *foxp3*, *IL-10*, and *TGF-β* (Fig. 6) while negatively correlated with Th17 cell-related transcriptional factors and cytokines such as *rorγt*, *Stat3*, *IL-6*, *IL-17A*, and *IL-21* (Fig. 6). On the other hand, *Enterococcus* and *Streptococcus*, the opportunistic pathogenic bacteria, were negatively correlated with growth performance as well as Treg cell-associated transcriptional factors and cytokines and positively correlated with Th17 cell-related transcriptional factors and cytokines (Fig. 6).

## Discussion

Th17 and Treg cells are differentiated from CD4^+^ T cells and are synergistically implicated in gut immunological functions [29]. It has been established that Th17/Treg cell balance is crucial in maintaining intestinal immune homeostasis because Th17 cells are often implicated in autoimmunity/inflammation, whereas Treg cells work reciprocally [22, 29]. Altering Th17 and/or Treg cell differentiation leads to the perturbative intestinal homeostasis, which is attributed to the imbalanced Th17/Treg cells [30]. Another study reported that pathogenic Th17 cells suppress Treg generation and contribute to the development of inflammation [31]. Thus, imbalanced Th17/Treg cells augment the intensity of systemic inflammation, which impairs nutrient digestion and absorption, causing a reduction in chicken body weight gain [11, 32]. Scientists found that *IL-6*-mediated *Stat3* acts as an essential transcription factor for the differentiation of Th17 cells by inducing nuclear receptor, *rorγt* [33], whereas *TGF-β* stimulates *foxp3* transcription, which subsequently promotes Treg cell differentiation [18]. Elevated Th17 cells are associated with the pathogenesis of inflammatory bowel disease (IBD) and colitis through expressing *rorγt*, *IL-17A*, *IL-17F*, and *IL-21* in the inflamed mucosa [34, 35]. It is demonstrated that in colitis pathology, both *IL-17A* and *IL-17F* are involved in developing intestinal inflammation because these cytokines have overlapping or interdependent pro-inflammatory role [5, 36]. However, Treg cell-associated transcription factor *foxp3* restricts the polarization of Th17 cells by inhibiting their transcriptional potential [33], indicating an intricate interaction between these cells. Recent evidence revealed that *foxp3* and its associated cytokines, *TGF-β* and *IL-10*, were observed in maintaining Th17/Treg cell balance in the chicken gut [37, 38]. In the present study, upregulated mRNA expression of *IL-6*, *Stat3*, and *rorγt* in the jejunum of low body weight chickens of all three breeds indicated the higher differentiation of Th17 cells, and downregulated mRNA expression of *TGF-β* and *foxp3* in the jejunum of low body weight chickens of all three breeds indicated the lower differentiation of Treg cells. Typically, the relative mRNA expressions of *IL-17A* and *IL-21* were significantly higher in low body weight chickens, while the relative mRNA expression of *IL-10* was significantly lower in low body weight chickens, indicating imbalanced Th17/Treg cells result in intestinal inflammation and reduce chicken growth performance. Therefore, balancing intestinal Th17/Treg cells might be an effective way to improve chicken growth performance.

It has been established that gut microbiota is predominantly associated with intestinal immune homeostasis and prevents intestinal inflammation [19, 20]. Any disturbance in gut microbiota balance aggravates intestinal inflammation [39]. Recently, fecal microbiota transplantation (FMT) is emerging as a useful technique to reestablish the disturbed microbial communities and effectively contributes to the chicken growth [40]. Thus, in the current study, we elucidated whether FMT could reshape gut microbiota of the recipient chicks and increase their growth performance. Interestingly, we found that FMT significantly increased body weight, breast/leg muscle weight, and an average area of breast/leg muscles in the FMT group compared with the control group, indicating an association of microbiota transplantation with chicken growth. It is also well-known that chicken growth is related to the effective mucosal physiology in the intestine, which is dependent on such intestinal architecture that facilitates nutrient digestion, particularly villus height, providing enough surface area for nutrient absorption [41]. In the present study, markedly enhanced jejunal length and villus length in the jejunum by FMT indicated that transferring fecal suspension exerted beneficial effects on the intestinal architecture, resulting in enhanced nutrient digestion/absorption. Notably, *Lactobacillus*, a potential probiotic, is recognized as a predominant bacterium both in the human and animal gut. Its competitive exclusion greatly influences gut microbiota equilibrium, encouraging the useful bacteria, i.e., *Bifidobacteria*, yet discouraging the unfavorable bacteria, i.e., *Staphylococci*, *Escherichia coli*, or *Streptococci* [42, 43]. Consistent with these findings, we also observed a higher abundance of *Lactobacillus* in the jejunum of the FMT group, suggesting the growth-promoting potential of *Lactobacillus* in the chickens. On the other hand, a higher abundance of *Streptococcus* and *Enterococcus* in the control group of the present study indicated their inflammatory potential in the chicken gut because Steck and co-workers found *Enterococcus*-related-specific antibodies in the serum of inflammatory bowel disease patients and demonstrated that *Enterococcus* disrupted epithelial barrier, contributing to intestinal inflammation [44]. Accumulated evidence revealed that gut microbiota could regulate immune homeostasis by balancing Th17/Treg cells [21, 22]. Thus, next, we investigated whether FMT could modulate the associated transcriptional and cytokine parameters of Th17/Treg cells. In the present study, the upregulated relative mRNA expressions of *foxp3*, *TGF-β*, and *IL-10* in the jejunum of the FMT group and *rorγt*, *Stat3*, *IL-6*, *IL-17A*, and *IL-21* in the jejunum of control group indicate that early colonization of gut microbiota in the chicken jejunum maintains Th17/Treg cell balance by regulating their associated transcription factors and cytokines. Interestingly, our findings about Th17/Treg cell balance in the FMT group are consistent with high body weight chickens and reveal that transferring fecal microbiota could increase chicken growth performance by balancing Th17/Treg cells. However, whether other bacteria play synergistic action needs further study because there are some other bacteria besides *Lactobacillus* in the fecal suspension.

Recent evidence revealed that gut microbiota-derived metabolites maintain intestinal homeostasis by regulating the host immune system, mainly through balancing Th17/Treg cells [22, 45]. Typically, tryptophan has a key significance in the chickens because gut microbiota directly or indirectly influences tryptophan and transforms it into ligands of the aryl-hydrocarbon receptor (*AhR*), serotonin, and indole and/or its derivatives [45, 46], and *Lactobacilli* are the potential bacteria producing these metabolites [47, 48]. Fouad et al. reported that tryptophan is principally involved in encouraging the beneficial bacteria, i.e., *Lactobacilli* and *Bifidobacteria*, and inhibited the harmful bacteria, *Escherichia coli* [49]. In the present study, metabolite KEGG pathway enrichment map revealed that tryptophan metabolism was the most significant pathway, and serotonin, indole, and 5-methoxyindoleacetate were also significantly higher in the FMT group, indicating the role of these metabolites in chicken growth. Interestingly, our results are consistent with the recently reported findings demonstrating indole to strengthen the gut barrier functions by ameliorating the enteropathy [50] and serotonin in nutrients absorption and immune response [27]. Furthermore, Saeedi et al. also found that *Lactobacilli*-produced 5-methoxyindoleacetate protects liver toxicity by travelling from gut to the liver, facilitating hepatic metabolism [51]. On the other hand, another study demonstrated that tryptophan metabolites inhibited Th17 cell’s polarization towards a pro-inflammatory phenotype by reprogramming CD4^+^ cells into Treg cells [52]. Particularly, serotonin promotes Treg cells while inhibits Th17 cells [53], and indole also regulates these cells and protects intestine from tissue injury via *AhR* activation in chickens [5]. In the present study, a higher *Lactobacillus* abundance along with prominently elevated tryptophan metabolites (serotonin, indole, and 5-methoxyindoleacetate) and Treg cells in the FMT group suggested a cumulative effect on increased chicken growth performance by increasing nutrient absorption and immune regulation, which are distinctly supported by the above described findings. It has also been described that *AhR* activation is crucial in maintaining intestinal homoeostasis. After binding with the ligand, *AhR* translocates to the nucleus and binds with *AhR* nuclear translocator, forming a heterodimer, which modulates the target gene (*CYP1A2*) expression through binding with xenobiotic-responsive elements [54, 55]. It is also found that downregulation of *CYP1A2* is observed during inflammation [56], whereas *AhR* activation protects intestinal epithelium from enterocolitis by promoting *CYP1A2* expression [57–59], and that indole derivatives activate *AhR* to promote *IL-22* secretion, which boosts intestinal mucosal defense [60]. Interestingly, the present study found a significantly elevated expression of *AhR*, *CYP1A2*, and *IL-22* in the jejunum of the FMT group, indicating that gut microbiota (probably *Lactobacillus*)-produced metabolites protect jejunum from inflammation and promote intestinal homeostasis. These findings reveal that gut microbiota-produced metabolites enhance chicken growth by balancing Th17/Treg cells.

## Conclusions

Taken together, these findings suggested that imbalanced Th17/Treg cells decreased chicken growth performance. On the other hand, FMT-reshaped gut microbiota produced more tryptophan and its metabolites including serotonin, indole, and 5-methoxyindoleacetate, which activated aryl-hydrocarbon receptor (*AhR*) and expressed more *CYP1A2* and *IL-22* to maintain Th17/Treg cell balance and immune homeostasis and finally increased chicken growth performance.

## Materials and methods

### Animals

The approval for all the procedures related to animal’s experimentation was obtained from Institutional Animal Care and Use Committee of Huazhong Agricultural University (HZAUCH-2018-008), and all methods are conducted as permitted by the relevant guidelines and regulations.

In order to get the chickens with significantly different growth performance, newly hatched chicks including Turpan cockfighting × White Leghorn chickens, white feather chickens, and yellow feather chickens were used in the present study. A total of 600 chickens (200 chickens for each breed) were used in the current study. The chickens of all three breeds were raised in the metallic cages (5 chickens per cage) under standard housing conditions in the poultry farm at Huazhong Agricultural University. All chickens have free access to food and water, were reared without vaccinations or medications, and were fed pelleted diet of corn and soybeans. At 6 weeks of age, all birds were weighed, and ten male chickens with the highest body weight and ten male chickens with the lowest body weight were selected from each chicken breed respectively for further research. For the fecal microbiota transplantation (FMT) experiment, 60 1-day-old male yellow feather chicks with the same genetic background were selected as recipients.

### Selection of FMT donors

In order to get the good FMT donors, in the Turpan cockfighting × White Leghorn chickens (3 months old), four chickens (two males and two females) with high body weight and other four chickens (two males and two females) with low body weight were selected. The microbiota in the feces was compared using 16S rRNA gene sequencing. The results showed that the relative abundance of *Lactobacillus* in high body weight chickens was significantly higher compared with low body weight chickens. Besides, the relative abundance of *Lactobacillus* in female high body weight chickens was significantly higher than that of male chickens (Fig. S1A). Moreover, the fecal bacterial composition of our selected donor chickens showed stable changes at different weeks (Fig. S1B), indicating that it could provide a stable source of bacteria for the recipient chickens during the 4 weeks of the FMT experiment. Therefore, the female high body weight chicken with the most abundant *Lactobacillus* was selected as the FMT donor.

### Preparation of fecal suspensions

Every morning, once the donor chicken defecated, the white part of the fecal materials was removed as it contains uric acid. Then, 7 g of fecal materials was collected in 50-mL sterile centrifuge tube and mixed with saline (0.75%) in a ratio of 1:6 (mixing 6 mL of saline per gram of feces). The mixer in the sterile tube was then placed in the ice box until the residue completely settled. Subsequently, after collecting the supernatant, it was filtered using a germ-free gauze, and fecal suspension was obtained.

### Animal treatment

A total of 60 1-day-old male yellow feather chicks were selected as recipients and randomly divided into control group and FMT group (*n* = 30). Chickens in the FMT group orally received fecal suspension (1 mL), and chickens in the control group orally received 0.75% saline (1 mL) every afternoon for 28 days. All chickens were sacrificed at the 30th day, and samples were collected for subsequent analysis.

### Samples collection

After 12 h of fasting, all chickens were weighed and sacrificed. The weights of the leg and breast muscles were determined, and jejunal length was also measured. For analysis of gut microbiota and untargeted metabolomic profiles, 13 to 15 cm jejunal segments from each bird were rapidly excised, and the content (1 to 1.5 g per bird) was collected into two sterilized centrifuge tubes (1.5 mL), quickly snap-frozen in liquid nitrogen, and then stored at −80 °C for sequencing. For histomorphological analysis, freshly harvested muscle and jejunum tissues were fixed in 4% paraformaldehyde solution. For gene expression analysis, the harvested jejunal segments were cut open, gently flushed with 0.75% normal-saline, frozen immediately in the liquid nitrogen, and then stored in −80 °C refrigerator.

### Hematoxylin and eosin staining

For morphological observation, leg muscle, breast muscle, and jejunum tissue samples were embedded in paraffin and were cut into 3-µm-thick sections using a rotary sectioning machine (LEICARM2245, Leica, Germany). Finally, the tissue sections were stained with standard hematoxylin-eosin staining following the steps in our previously described work [2].

### Immunohistochemical staining

The protein expression and distribution of *foxp3*, *rorγt*, and *AhR* in jejunum were detected by immunohistochemical staining according to the steps of our previously published literature [2]. For the jejunal sections, deparaffinization was performed in the xylene, and hydration was carried out with gradient ethanol solutions. After this, to repair the antigen, the sections were then placed in citrate buffer solution, and after warming in the microwave oven, these sections were put at room temperature for cooling down. To inactivate the endogenous peroxidase, 3% hydrogen peroxide was applied, and nonspecific binding sites were blocked by incubating the tissue sections in 5% BSA for 30 min at 37 °C. The primary antibodies of rabbit anti-*foxp3* (1:300) (A12685, Abclonal, China), the rabbit anti-*rorγt* (1:300) (A10240, Abclonal, China), and rabbit anti-*AhR* (1:300) (A1451, Abclonal, China) were then added drop by drop to the sections and incubated at 4 °C for 12 h. Consequently, secondary antibodies (horseradish peroxidase) were then added dropwise to the tissue section and incubated at 37 °C for half an hour. Finally, DAB chromogenic method was used to develop the tissue sections and then re-stained with hematoxylin, followed by sealing the sections.

### Quantitative real-time polymerase chain reaction (qPCR)

To detect the relative mRNA expression levels of genes associated with Th17 and Treg cells, the extraction of total RNA from jejunal segments was performed with the TRIzol reagents (Takara, Japan) following manufacturer instructions. After removing genome DNA, RNA (1 µg) from every sample was reverse-transcribed to get cDNA with PrimeScript™-RT reagent Kit with (gDNA) Eraser (Takara, Japan). A total of 10-μL qPCR reaction mixer contains SYBR dye (5 µL) (Takara, Japan); enzyme-free water (3.2 µL); forward primer (0.4 µL); reverse primer (0.4 µL); and template cDNA (1 µL). A real-time qPCR-probing system (Bio-Rad CFX Connect) was used to perform qPCR reaction (Bio-Rad, Hercules, CA, USA). The steps are as follows: 5 min predenaturation at 95 °C, 30 s denaturation (40 cycles) at 95 °C, 30 s annealing at 60 °C, and finally 15 s extension at 72 °C. The sequences of different primers were listed in Table 1 following a reference gene (*β-actin*). Using 2^−ΔΔCT^ method, the expression levels of different genes were quantitatively calculated.

**Table 1:** The sequences of different primers for qPCR

**Genes**	**Primer sequence (5′ to 3′)**	**Accession no.**
*β-actin*	f-TTGTTGACAATGGCTCCGGT	NM_205518.2
	r-TCTGGGCTTCATCACCAACG	
*foxp3*	f-AACGGCGAGACACCTTC	NM_001024827.2
	r-TTCGGAGACTTTAATCCACTA	
*IL-10*	f-AGATGCTGCGCTTCTACACA	NM_001004414.4
	r-CCCATGCTCTGCTGATGACT	
*TGF-β*	f-ATGTGTTCCGCTTTAACGTGTC	NM_205454.2
	r-GCTGCTTTGCTATATGCTCATC	
*rorγt*	f-CACCCCCAGCTTCACCATAG	XM_040691084.2
	r-GCAGCTCAATCTCCAATGCG	
*Stat3*	f-GCCGAATCACAACTACAGACTC	NM_001030931.4
	r-CTGACTTTGGTGGTGAACTGC	
*IL-6*	f-CTCCTCGCCAATCTGAAGTC	NM_204628.2
	r-AGGCACTGAAACTCCTGGTCT	
*IL-17F*	f-TTGACATTCGCATTGGCAGC	XM_040668476.2
	r-AGTTCAAGCAGCCCAAGAGG	
*IL-17A*	f-AAGGTGATACGGCCAGGACT	NM_204460.2
	r-GAGTTCACGCACCTGGAATG	
*IL-21*	f-TCTGTTCAGTGACTTGCCCC	NM_001024835.2
	f-CCAACCACCCTTTAGCCACT	
*AhR*	f-ACCTGTGCAGAAAATAGTAAAGCC	NM_204118.3
	r-CTTCCAGGATCTGCATCCCC	
*CYP1A2*	f-TGGATACCCTCTGCCTCTCTC	NM_205146.3
	r-CTAAGGGGAAGCGTGGTGTA	
*IL-22*	f-GCCCTACATCAGGAATCGCA	NM_001199614.1
	r-CCACATCCTCAGCATACGGG	

### Non-targeted metabolomics

#### Extraction and determination of metabolites

Jejunal contents (50 mg) were collected, and metabolite extraction was performed using a solution (4:1, v/v) of 400-µL methanol: water and L-2-chlorophenylalanin (0.02 mg/mL) was used as a reference standard. After settling this mixture at −10 °C, it was treated with 50 Hz for 6 min using a high-throughput tissue-crusher Wonbio 96c (Shanghai Wanbo Biotechnology Co., Ltd.) following performing an ultrasound for 30 min at 5 °C and 40 kHz. The precipitation of proteins was performed by placing the samples at 20 °C. After this, the centrifugation (13,000 g) of the samples was performed for 15 min at 4 °C. The supernatant solutions were obtained and cautiously transferred to sample vials for subsequent LC-MS analysis. Quality-controlled samples are prepared by combining all samples in equal parts with good reproducibility.

The platform of UHPLC-Q Exactive HF-X system (ThermoFisher Scientific) was used for LC–MS analysis. Briefly, HSS T3 column with dimensions (1.8 μm, 100 mm × 2.1 mm) was utilized to separate a 2-µL sample and then loaded into the mass-spectrometry detection system. The mobile phase contains formic acid (0.1%) in the solvent A (water:acetonitrile (95:5)v/v and formic acid (0.1%) in the solvent B (acetonitrile:isopropanol:water (47.5:47.5:5)v/v. The solvent gradients are as follows: from 0 to 0.1 min, 0 to 5% B; from 0.1 to 2 min, 5 to 25% B; from 2 to 9 min, 25 to 100% B; from 9 to 13 min, 100 to 100% B; from 13 to 13.1 min, 100 to 0% B; and from 13.1 to 16 min, 0 to 0% B for equilibrating the systems. A total of 2 µL of sample is applied at a time, and the flow rate is set to 0.4 mL per minute. The temperature of the column was 40 °C. The samples were stored in the 4 °C refrigerator during the experiment. The mass spectrometric data was collected using a Thermo UHPLC-Q Exactive Mass Spectrometer equipped with an electrospray ionization (ESI) source operating in either positive or negative ion mode.

#### Differential metabolites analysis

After obtaining the raw data from LC-MS, it was preprocessed using Progenesis QI (Waters Corporation, Milford, USA) software. Data is analyzed through majorbio-cloud platform (cloud.majorbio.com), and the variance analysis was performed on the obtained matrix file after preprocessing the sample data. The partial least squares-discriminant analysis (PLS-DA) and principal component analysis (PCA) were analyzed using the R package ropls (Version 1.6.2). A fold-difference analyses and the Student *t*-test were also performed. Variable importance in projection (VIP) was obtained after PLS-DA analysis and Student test, and based on the VIP values, the significant different metabolites were selected. The metabolites with VIP more than 1 and (*P* < 0.05) were considered as significantly differential metabolites. Based on the KEGG database (KEGG, http://www.genome.jp/kegg/), the differential metabolites between the two groups were mapped to biochemical metabolic pathways and the pathway enrichment analysis was also performed.

### 16S rRNA genes sequencing and analysis

The genomic DNA of microbial communities was extracted from the jejunal content with FastDNA-SPIN-extraction kit (MP Biomedicals, Santa Ana, CA, USA) following standard manufacturer’s instructions. Quantifications of the extracted DNA with OD (260/280) ranged from 1.8 to 2.0 were performed with NanoDrop ND1000 Spectrophotometer (ThermoFisher Scientific, Waltham, MA, USA) and with 1% agarose gel electrophoresis. Bacterial 16S-rRNA genes contain hypervariable regions (V3 and V4), which were amplified using forward 338F (5′-ACTCCTACGGGAGGCAGCA-3′) and reverse 806R (5′-GGACTACHVGGGTWTCTAAT-3′) primers, respectively, following steps in our previous work [2]. Purified amplicon library from every jejunal sample has 0.5 ng/µL concentrations. The pooling of purified amplicons in equimolar and pair-end sequencings (2 × 300 bp) was performed using the platform of Illumina MiSeq (Illumina, San Diego, USA) following standardized instructions from Majorbio Bio-Pharm Technology Co., Ltd. (Shanghai, People’s Republic of China). A total of 30,000 clean reads per purified-amplicon library were obtain using this method.

The raw reads from the 16S rRNA genes sequencing data were demultiplexed, filtered with Trimmomatic to get the quality reads and finally merged using FLASH. The clustering of operational taxonomic unit (OTU) using 97% similarity cutoff was performed with UPARSE (version 7.1, http://drive5.com/uparse/). Then, the valid reads were obtained after identifying and removing the chimeric sequence. To analyze taxonomic nature of every OTU-representative sequence against Silva 138 (16S rRNA) database, RDP Classifier (http://rdp.cme.msu.edu/) was applied following 0.7 as a confidence threshold.

In order to reduce the effect of sequencing depths on *α*- and *β*-diversity measurements, subsampling of reads from every sample was performed. The lowest effective reads for the jejunum contents of FMT and control groups are 42,687. The Shannon index as well as the Chao index was used to describe alpha diversity. Pie charts and bar charts were used to show the genus-level community structure. Based on default parameters, linear discriminant analysis effect size (LEfSe) was performed to check the taxa with significant differences between groups. Based on the Tax4Fun, sequence annotations were carried out using KEGG functions, and finally, STAMP software package was used to visualize the statistics of these sequences.

### Statistical analysis

The digital images of each sample were taken with the light microscope (BH-2, Olympus, Japan) using a digital camera (DP72, Olympus). From each group, 8 sections were selected, and 8 random field images were acquired in each section depending on the type of tissue. In each field of view image, the mean cross-sectional area of the single leg and/or breast muscle cell as well as the IHC-positive signal was counted using Image-Pro Plus 6.0 (Media Cybernetics, USA). The IOD-positive signal in each field of view and the length of jejunum villi were also determined. Prism software 8 (GraphPad, Inc., San Diego, USA) was applied to get the data graphics and analyses of the data. The data with means ± SEM are shown here, and the Student *t*-test was applied to determine the statistical significance. To compare mean between two groups, the value (*P* < 0.05) was used as a statistically significant value. In addition, metabolites with the variable importance in the projection (VIP) value of the PLS-DA model > 1 and the *P*-values of Student’s *t*-test < 0.05 were considered to be significantly different.

## Supplementary Information

**Additional file 1: Supplementary Fig. S1.** The fecal microbial composition of candidate donor chickens at the genus level. A. The fecal microbial composition of different donor chickens. B. The fecal microbial composition of the selected donor chicken at different time. HF represents female donor chickens with high body weight; HM represents male donor chickens with high body weight; LF represents female donor chickens with low body weight; LM represents male donor chickens with low body weight; W1-W4 represents one week, two weeks, three weeks, four weeks, respectively.

## Data Availability

The raw 16S rRNA gene and metagenomic sequencing data are available at the NCBI Sequence Read Archive (SRA), under BioProject (PRJNA900164).

## References

[1] Garcia-Carbonell R, Yao SJ, Das S, Guma M (2019). Dysregulation of intestinal epithelial cell RIPK pathways promotes chronic inflammation in the IBD gut. Front Immunol.

[2] Zhang X, Akhtar M, Chen Y, Ma Z, Liang Y, Shi D (2022). Chicken jejunal microbiota improves growth performance by mitigating intestinal inflammation. Microbiome.

[3] Villablanca EJ, Selin K, Hedin CRH (2022). Mechanisms of mucosal healing: treating inflammatory bowel disease without immunosuppression?. Nat Rev Gastroenterol Hepatol.

[4] Ahmad R, Yu YH, Hsiao FS, Su CH, Liu HC, Tobin I (2022). Influence of heat stress on poultry growth performance, intestinal inflammation, and immune function and potential mitigation by probiotics. Animals (Basel).

[5] Kim WH, Lillehoj HS, Min W (2019). Indole treatment alleviates intestinal tissue damage induced by chicken coccidiosis through activation of the aryl hydrocarbon receptor. Front Immunol.

[6] Cardoso Dal Pont G, Lee A, Bortoluzzi C, Farnell YZ, Gougoulias C, Kogut MH (2023). Novel model for chronic intestinal inflammation in chickens: (2) immunologic mechanism behind the inflammatory response. Dev Comp Immunol.

[7] Lin Z-X, Zhang M, Yang R, Min Y, Guo P-T, Zhang J (2023). The anti-inflammatory effect of lutein in broilers is mediated by regulating TLR4/MyD88 signaling pathway. Poult Sci.

[8] Terada T, Nii T, Isobe N, Yoshimura Y (2020). Effect of antibiotic treatment on microbial composition and expression of antimicrobial peptides and cytokines in the chick cecum. Poult Sci.

[9] Strober W, Zhang F, Kitani A, Fuss I, Fichtner-Feigl S (2010). Proinflammatory cytokines underlying the inflammation of Crohn’s disease. Curr Opin Gastroenterol.

[10] Qiu X, Zhang M, Yang X, Hong N, Yu C (2013). Faecalibacterium prausnitzii upregulates regulatory T cells and anti-inflammatory cytokines in treating TNBS-induced colitis. J Crohns Colitis.

[11] Song B, Li P, Yan S, Liu Y, Gao M, Lv H (2022). Effects of dietary astragalus polysaccharide supplementation on the Th17/Treg balance and the gut microbiota of broiler chickens challenged with necrotic enteritis. Front Immunol.

[12] Shahini A, Shahini A. Role of interleukin-6-mediated inflammation in the pathogenesis of inflammatory bowel disease: focus on the available therapeutic approaches and gut microbiome. J Cell Commun Signal. 2023;17:55–74. 10.1007/s12079-022-00695-x. 10.1007/s12079-022-00695-x PMC10030733 36112307

[13] Gong Y, Lin Y, Zhao N, He X, Lu A, Wei W (2016). The Th17/Treg immune imbalance in ulcerative colitis disease in a Chinese Han population. Mediators Inflamm.

[14] Yan J-b, Luo M-m, Chen Z-y, He B-h (2020). The function and role of the Th17/Treg cell balance in inflammatory bowel disease. J Immunol Res.

[15] Zhou L, Ivanov II, Spolski R, Min R, Shenderov K, Egawa T (2007). IL-6 programs TH-17 cell differentiation by promoting sequential engagement of the IL-21 and IL-23 pathways. Nat Immunol.

[16] Coccia M, Harrison OJ, Schiering C, Asquith MJ, Becher B, Powrie F (2012). IL-1β mediates chronic intestinal inflammation by promoting the accumulation of IL-17A secreting innate lymphoid cells and CD4(+) Th17 cells. J Exp Med.

[17] Fina D, Sarra M, Fantini MC, Rizzo A, Caruso R, Caprioli F (2008). Regulation of gut inflammation and th17 cell response by interleukin-21. Gastroenterology.

[18] Zhang H, Caudle Y, Wheeler C, Zhou Y, Stuart C, Yao B (2018). TGF-β1/Smad2/3/Foxp3 signaling is required for chronic stress-induced immune suppression. J Neuroimmunol.

[19] Yoo JY, Groer M, Dutra SVO, Sarkar A, McSkimming DI (2020). Gut microbiota and immune system interactions. Microorganisms.

[20] Akhtar M, Chen Y, Ma Z, Zhang X, Shi D, Khan JA (2022). Gut microbiota-derived short chain fatty acids are potential mediators in gut inflammation. Anim Nutr.

[21] López P, de Paz B, Rodríguez-Carrio J, Hevia A, Sánchez B, Margolles A (2016). Th17 responses and natural IgM antibodies are related to gut microbiota composition in systemic lupus erythematosus patients. Sci Rep.

[22] Cheng H, Guan X, Chen D, Ma W (2019). The Th17/Treg cell balance: a gut microbiota-modulated story. Microorganisms.

[23] Lin L, Zhang J (2017). Role of intestinal microbiota and metabolites on gut homeostasis and human diseases. BMC Immunol.

[24] Chen X, Chen W, Ci W, Zheng Y, Han X, Huang J, et al. Effects of dietary supplementation with Lactobacillus acidophilus and Bacillus subtilis on mucosal immunity and intestinal barrier are associated with its modulation of gut metabolites and microbiota in late-phase laying hens. Probiotics Antimicrob Proteins. 2022. 10.1007/s12602-022-09923-7. 10.1007/s12602-022-09923-7 35138584

[25] Metzler-Zebeli BU, Siegerstetter SC, Magowan E, Lawlor PG, O’Connell NE, Zebeli Q (2019). Fecal microbiota transplant from highly feed efficient donors affects cecal physiology and microbiota in low- and high-feed efficient chickens. Front Microbiol.

[26] Glendinning L, Chintoan-Uta C, Stevens MP, Watson M (2022). Effect of cecal microbiota transplantation between different broiler breeds on the chick flora in the first week of life. Poult Sci.

[27] Fu Y, Hu J, Erasmus MA, Johnson TA, Cheng HW (2022). Effects of early-life cecal microbiota transplantation from divergently selected inbred chicken lines on growth, gut serotonin, and immune parameters in recipient chickens. Poult Sci.

[28] Liu YJ, Tang B, Wang FC, Tang L, Lei YY, Luo Y (2020). Parthenolide ameliorates colon inflammation through regulating Treg/Th17 balance in a gut microbiota-dependent manner. Theranostics.

[29] Lee GR (2018). The balance of Th17 versus Treg cells in autoimmunity. Int J Mol Sci.

[30] Ai TL, Solomon BD, Hsieh CS (2014). T-cell selection and intestinal homeostasis. Immunol Rev.

[31] Yoo SA, Kim M, Kang MC, Kong JS, Kim KM, Lee S (2019). Placental growth factor regulates the generation of T(H)17 cells to link angiogenesis with autoimmunity. Nat Immunol.

[32] Tan J, Liu S, Guo Y, Applegate TJ, Eicher SD (2014). Dietary L-arginine supplementation attenuates lipopolysaccharide-induced inflammatory response in broiler chickens. Br J Nutr.

[33] Chung Y, Chang SH, Martinez GJ, Yang XO, Nurieva R, Kang HS (2009). Critical regulation of early Th17 cell differentiation by interleukin-1 signaling. Immunity.

[34] Tsuji NM, Kosaka A (2008). Oral tolerance: intestinal homeostasis and antigen-specific regulatory T cells. Trends Immunol.

[35] Lee SH, Kwon JE, Cho ML (2018). Immunological pathogenesis of inflammatory bowel disease. Intest Res.

[36] Wedebye Schmidt EG, Larsen HL, Kristensen NN, Poulsen SS, Lynge Pedersen AM, Claesson MH (2013). TH17 cell induction and effects of IL-17A and IL-17F blockade in experimental colitis. Inflamm Bowel Dis.

[37] Tong B, Yu J, Wang T, Dou Y, Wu X, Kong L (2015). Sinomenine suppresses collagen-induced arthritis by reciprocal modulation of regulatory T cells and Th17 cells in gut-associated lymphoid tissues. Mol Immunol.

[38] Yu M, Meng T, He W, Huang H, Liu C, Fu X (2021). Dietary chito-oligosaccharides improve intestinal immunity via regulating microbiota and Th17/Treg balance-related immune signaling in piglets challenged by enterotoxigenic E. coli. J Agric Food Chem.

[39] Matsuoka K, Kanai T (2015). The gut microbiota and inflammatory bowel disease. Semin Immunopathol.

[40] Zhang X, Hu Y, Ansari AR, Akhtar M, Chen Y, Cheng R (2022). Caecal microbiota could effectively increase chicken growth performance by regulating fat metabolism. Microb Biotechnol.

[41] Awad W, Ghareeb K, Böhm J (2008). Intestinal structure and function of broiler chickens on diets supplemented with a synbiotic containing Enterococcus faecium and oligosaccharides. Int J Mol Sci.

[42] Forte C, Manuali E, Abbate Y, Papa P, Vieceli L, Tentellini M (2018). Dietary Lactobacillus acidophilus positively influences growth performance, gut morphology, and gut microbiology in rurally reared chickens. Poult Sci.

[43] Roy K, Bertelsen MF, Pors SE, Johansen KW, Kristensen AT, Kjelgaard-Hansen M (2014). Inflammation-induced haemostatic response in layer chickens infected with Streptococcus equi subsp. zooepidemicus as evaluated by fibrinogen, prothrombin time and thromboelastography. Avian Pathol.

[44] Steck N, Hoffmann M, Sava IG, Kim SC, Hahne H, Tonkonogy SL (2011). Enterococcus faecalis metalloprotease compromises epithelial barrier and contributes to intestinal inflammation. Gastroenterology.

[45] Khan S, Moore RJ, Stanley D, Chousalkar KK (2020). The gut microbiota of laying hens and its manipulation with prebiotics and probiotics to enhance gut health and food safety. Appl Environ Microbiol.

[46] Agus A, Planchais J, Sokol H (2018). Gut microbiota regulation of tryptophan metabolism in health and disease. Cell Host Microbe.

[47] O’Mahony SM, Clarke G, Borre YE, Dinan TG, Cryan JF (2015). Serotonin, tryptophan metabolism and the brain-gut-microbiome axis. Behav Brain Res.

[48] Heeney DD, Gareau MG, Marco ML (2018). Intestinal Lactobacillus in health and disease, a driver or just along for the ride?. Curr Opin Biotechnol.

[49] Fouad AM, El-Senousey HK, Ruan D, Wang S, Xia W, Zheng C (2021). Tryptophan in poultry nutrition: impacts and mechanisms of action. J Anim Physiol Anim Nutr.

[50] Takáčová M, Bomba A, Tóthová C, Micháľová A, Turňa H (2022). Any future for faecal microbiota transplantation as a novel strategy for gut microbiota modulation in human and veterinary medicine?. Life (Basel).

[51] Saeedi BJ, Liu KH, Owens JA, Hunter-Chang S, Camacho MC, Eboka RU (2020). Gut-resident lactobacilli activate hepatic Nrf2 and protect against oxidative liver injury. Cell Metab.

[52] Quraishi MN, Shaheen W, Oo YH, Iqbal TH (2020). Immunological mechanisms underpinning faecal microbiota transplantation for the treatment of inflammatory bowel disease. Clin Exp Immunol.

[53] Wan M, Ding L, Wang D, Han J, Gao P (2020). Serotonin: a potent immune cell modulator in autoimmune diseases. Front Immunol.

[54] Sulem P, Gudbjartsson DF, Geller F, Prokopenko I, Feenstra B, Aben KK (2011). Sequence variants at CYP1A1-CYP1A2 and AHR associate with coffee consumption. Hum Mol Genet.

[55] Larigot L, Juricek L, Dairou J, Coumoul X (2018). AhR signaling pathways and regulatory functions. Biochim Open.

[56] Alhouayek M, Gouveia-Figueira S, Hammarström M-L, Fowler CJ (2018). Involvement of CYP1B1 in interferon γ-induced alterations of epithelial barrier integrity. Br J Pharmacol.

[57] Postal BG, Ghezzal S, Aguanno D, André S, Garbin K, Genser L (2020). AhR activation defends gut barrier integrity against damage occurring in obesity. Mol Metab.

[58] Lu P, Yamaguchi Y, Fulton WB, Wang S, Zhou Q, Jia H (2021). Maternal aryl hydrocarbon receptor activation protects newborns against necrotizing enterocolitis. Nat Commun.

[59] Hu N, Huang Y, Gao X, Li S, Yan Z, Wei B (2017). Effects of dextran sulfate sodium induced experimental colitis on cytochrome P450 activities in rat liver, kidney and intestine. Chem Biol Interact.

[60] Taleb S (2019). Tryptophan dietary impacts gut barrier and metabolic diseases. Front Immunol.

