# Supplementary material for: Fecal microbiota transplantation improves chicken growth performance by balancing jejunal Th17/Treg cells
Source: Microbiome. 2023 Jun 21;11:137. doi: 10.1186/s40168-023-01569-z (PMC10283253; doi:10.1186/s40168-023-01569-z)
Supplement: Supplementary file 2 — Additional file 1: Supplementary Fig. S1. The fecal microbial composition of candidate donor chickens at the genus level. A. The fecal microbial composition of different donor chickens. B. The fecal microbial composition of the selected donor chicken at different time. HF represents female donor chickens with high body weight; HM represents male donor chickens with high body weight; LF represents female donor chickens with low body weight; LM represents male donor chickens with low body weight; W1-W4 represents one week, two weeks, three weeks, four weeks, respectively. [file 40168_2023_1569_MOESM1_ESM.docx]

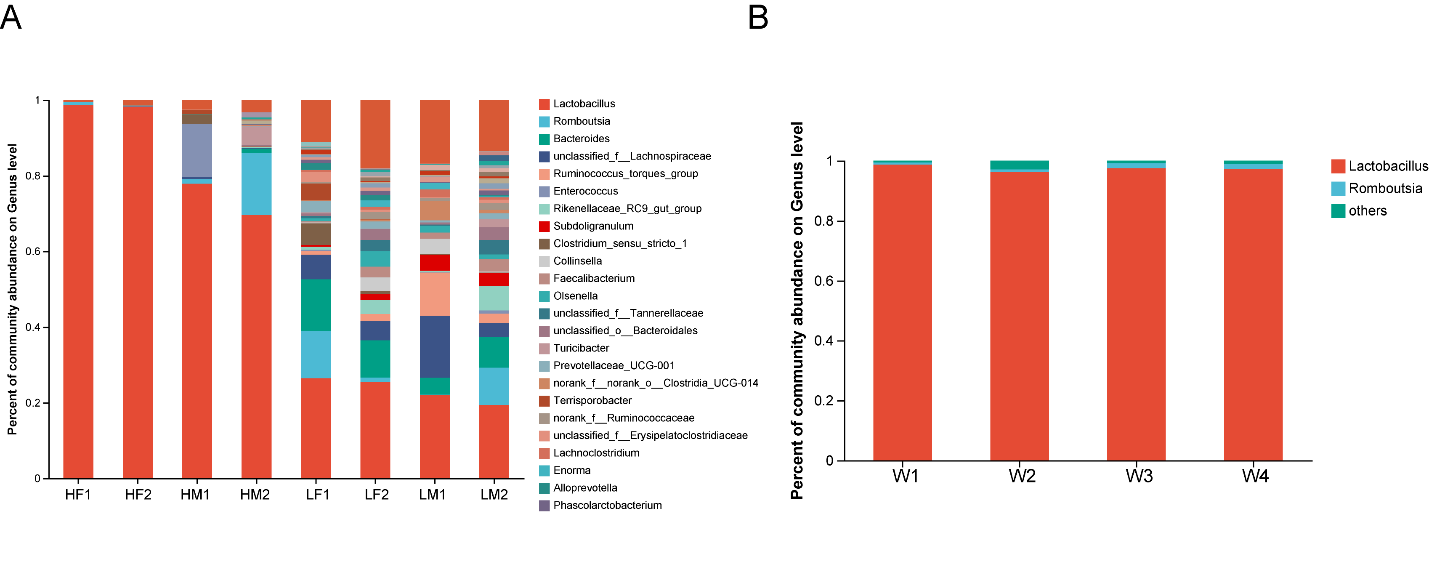
**Supplementary figure**

**Supplementary figure S1 (Fig. S1) The fecal microbial composition of candidate donor chickens at the genus level.**

A. The fecal microbial composition of different donor chickens; B. The fecal microbial composition of the selected donor chicken at different time. HF represents female donor chickens with high body weight; HM represents male donor chickens with high body weight; LF represents female donor chickens with low body weight; LM represents male donor chickens with low body weight; W1-W4 represents one week, two weeks, three weeks, four weeks, respectively.
